# Comparative Analysis of VOCs in Exhaled Breath of Amyotrophic Lateral Sclerosis and Cervical Spondylotic Myelopathy Patients

**DOI:** 10.1038/srep26120

**Published:** 2016-05-23

**Authors:** Changsong Wang, Mingjuan Li, Hongquan Jiang, Hongshuang Tong, Yue Feng, Yue Wang, Xin Pi, Lei Guo, Maomao Nie, Honglin Feng, Enyou Li

**Affiliations:** 1 Department of Anesthesiology, the First Affiliated Hospital of Harbin Medical University, Harbin, China; 2 Department of critical care medicine, the Third Affiliated Hospital of Harbin Medical University, Harbin, China; 3 Department of Neurology, the First Affiliated Hospital of Harbin Medical University, Harbin, China

## Abstract

Amyotrophic lateral sclerosis (ALS) is an incurable neurological degenerative disease. It can cause irreversible neurological damage to motor neurons; typical symptoms include muscle weakness and atrophy, bulbar paralysis and pyramidal tract signs. The ALS-mimicking disease cervical spondylotic myelopathy (CSM) presents similar symptoms, but analysis of breath volatile organic compounds (VOCs) can potentially be used to distinguish ALS from CSM. In this study, breath samples were collected from 28 ALS and 13 CSM patients. Subsequently, gas chromatography/mass spectrometry (GCMS) was used to analyze breath VOCs. Principal component analysis (PCA) and orthogonal partial least-squares discriminant analysis (OPLSDA) were the statistical methods used to process the final data. We identified 4 compounds with significantly decreased levels in ALS patients compared with CSM controls: (1) carbamic acid, monoammonium salt; (2) 1-alanine ethylamide, (S)-; (3) guanidine, N,N-dimethyl-; and (4) phosphonic acid, (p-hydroxyphenyl)-. Currently, the metabolic origin of the VOCs remains unclear; however, several pathways might explain the decreasing trends observed. The results of this study demonstrate that there are specific VOC profiles associated with ALS and CSM patients that can be used to differentiate between the two. In addition, these metabolites could contribute to a better understanding of the underlying pathophysiological mechanisms of ALS.

Amyotrophic lateral sclerosis (ALS), a known motor neuron disease, is a progressive neurological degenerative disease characterized by upper and lower motor neuron degeneration. Previous studies have shown that the incidence of ALS is approximately 1/100,000 in the general population and the prevalence is approximately 4/100,000 in Caucasians^1^. The disease is divided into sporadic ALS (sALS) and familial ALS (fALS)^2^. ALS is an incurable disease, and most ALS patients die within 2–5 years of the onset of symptoms owing to rapid progression, irreversible pathological changes, delayed diagnosis, lack of effective drugs, etc.^3^. Researchers have recommended treatment with Rilutek,the only effective medication for ALS (approved by the FDA in 1995), as early as possible to maximize the efficiency of the drug in preserving epibiotic motor neuron functions under the threat of irreversible neurological damage^4^. The typical clinical symptoms of ALS include muscle weakness and atrophy, bulbar paralysis and pyramidal tract signs. Likewise, patients with cervical spondylotic myelopathy (CSM) show many symptoms that mimic ALS, which frequently makes it challenging to distinguish between the two. Therefore, identifying a set of reliable biomarkers to distinguish ALS from CSM may help improve therapeutic effects as well as prognosis.

At present, the most important examinations used to distinguish ALS from CSM mainly include neurological examination, needle electromyography (EMG) and muscle biopsy^5^. However, neurological examination is usually a primary focus at disease onset. While the results of needle electromyography (EMG) and muscle biopsy are more accurate, these operations are invasive, which can cause discomfort for patients. Moreover, patients with early-stage ALS usually undergo several courses of needle EMG examination until the diagnosis is confirmed^6^. As a recently developed approach to screen for biomarkers of certain diseases, volatile organic compound (VOC) analysis in exhaled breath has attracted the increasing attention of researchers for its convenience, noninvasiveness, and the fact that it is well-tolerated by patients^7^,^8^.

The process of extracting valuable information and screening potential biomarkers from complex metabolomics data can directly affect the results of VOC analysis. In general, metabolomics data do not tend to meet the conditions of a parametric test. Therefore, some nonparametric test methods have commonly been used to determine the statistical significance of each metabolite, such as the Kruskal Wallis rank sum test, an extension of the Mann-Whitney U test^9^. The false discovery rate (FDR) is commonly used in multiple hypotheses testing to correct for multiple comparisons^10^. In addition, common multivariate statistical analysis methods in VOC analysis, such as PCA, partial least squares discriminant analysis (PLS-DA), OPLS-DA and artificial neural network (ANN), are often used to understand the metabolic differences among groups^11^,^12^. In this study, the Kruskal Wallis rank sum test, FDR, PCA and OPLS-DA were applied to process the final data.

In our previous studies, we identified 12 potential biomarkers of ALS in the blood samples of SOD1 G93A mice with a VOC platform^13^. The SOD1 G93A mouse used in our study was a transgenic mouse that has been widely applied to ALS research as a study tool.

In the current study, we analyzed the VOCs in exhaled breath samples from both ALS and CSM patients using solid phase microextraction-gas chromatography-mass spectrometry (SPME-GCMS) to explore the potential biomarkers for differentiating ALS from CSM.

## Materials and Methods

### Subjects

We obtained informed consent from all subjects prior to any experiment. All methods in this study were carried out in accordance with approved guidelines. All experimental protocols were approved by the Ethics Committee of the first affiliated hospital of Harbin Medical University (No. 201314). We selected 28 patients for the ALS group; these individuals were diagnosed with ALS according to the revised “EI Escorial criteria”^5^ and admitted to the Department of Neurology at the First Affiliated Hospital of Harbin Medical University between Jan. 2014 and Apr. 2015. In addition, 13 CSM patients were selected from the Department of Orthopedics of the First Affiliated Hospital of Harbin Medical University to comprise the CSM control group.

Table 1 shows the details of the patients. There were 18 males and 10 females in the ALS group, while the CSM control group included 6 males and 7 females. The mean ages of individuals in the ALS group and CSM control group were 58.6 y and 53.5 y, respectively, with standard deviations (SD) of 12.54 y and 8.13 y, respectively.

### Solid-Phase Microextraction (SPME)

The extraction mechanism adopted in this study employed 75 μm-thick carboxen/polydimethylsiloxane fibers (Bellefonte, USA). The method involved exposing the SPME fiber, which had been inserted into the vial, to the gaseous sample for 40 min with the temperature kept at 40 °C. Then, the hot Gas Chromatography (GC) injector was used for de-absorption of the volatiles, which proceeded for 2 min at 200 °C.

### Gas Chromatography-Mass Spectrometry (GCMS) Analysis

A GCMS (Shimadzu GCMS QP 2010, Shimadzu, Japan) equipped with a DB-5MS (length 30 m × ID 0.250 × film thickness 0.25 μm) (Agilent Technologies, USA) plot column was used for the sample analysis. The injector temperature was consistently maintained at 200 °C. The splitless mode was adopted for sample injections, with the flow rate of the helium (99.999%) carrier gas constantly kept at 2 ml min^−1^. To concentrate the hydrocarbons at the head of the column, it was necessary to maintain the temperature of the column at 40 °C for 1 min. Subsequently, the temperature of the column rose at a speed of 5 °C min^−1^ to 200 °C and lasted for 1 min; it then continued to rise to 230 °C at a speed of 15 °C min^−1^. The full-scan mode was used for MS analyses, with a scan range of 35–350 atomic mass unit (amu). Each measurement in this study was performed under the condition of 70 eV ionization energy and an ion source temperature of 230 °C.

### Extraction and Pretreatment of the GCMS Raw Data

Shimadzu GCMS Postrun Analysis software was used to convert raw GCMS data into CDF format (NetCDF) files, and the XCMS toolbox (http://metlin.scripps.edu/download/) was used for processing.

### Statistical Analysis

Total area normalization was performed for each sample before statistical analysis. The normalized data were then exported to the SIMCA-P 11.5 platform for PCA and OPLSDA. In this study, dual authentication standards of nonparametric tests and permutation tests were adopted^14^,^15^ to improve the credibility of the research results. To prevent the occurrence of overfitting, we performed the permutation tests with 100 iterations to validate the supervised model. Potential metabolic biomarkers were selected based on variable importance in the projection (VIP) values calculated from the PLS-DA model with a threshold of 1.0. In parallel, we applied the nonparametric Kruskal Wallis rank sum test to further validate the metabolites at a critical *p* value of 0.05^16^,^17^,^18^. Additionally, the FDR control was performed for multiple testing correction^10^. In our study, the FDR value was set at 0.1. To obtain the predicted probabilities of belonging to a group, a binary logistic regression was applied for the potential biomarkers. The receiver operating characteristic (ROC) curves were then constructed by plotting the sensitivity against 1-specificity. The area under the ROC curve (AUC) values were calculated to estimate the Accuracy of these potential biomarkers in distinguishing ALS from CSM.

## Results

### ALS Patients versus CSM Patients

**Figure 1:**
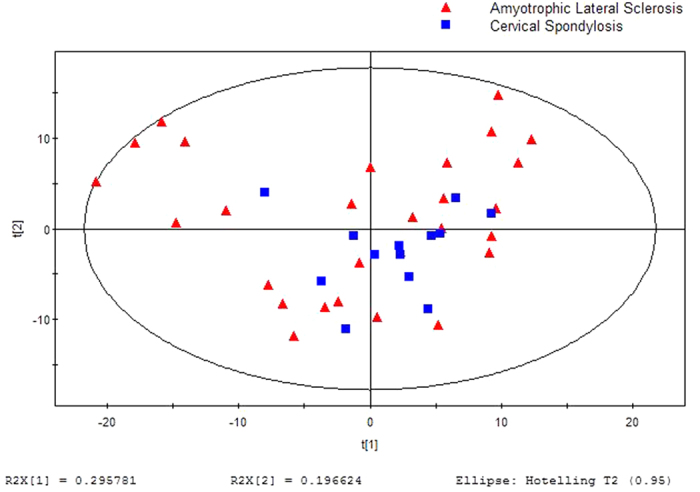
PCA score plot for breath samples from ALS Patients versus CSM Controls: (5 components, R^2^X = 0.750, Q^2^ = 0.580).

**Figure 2:**
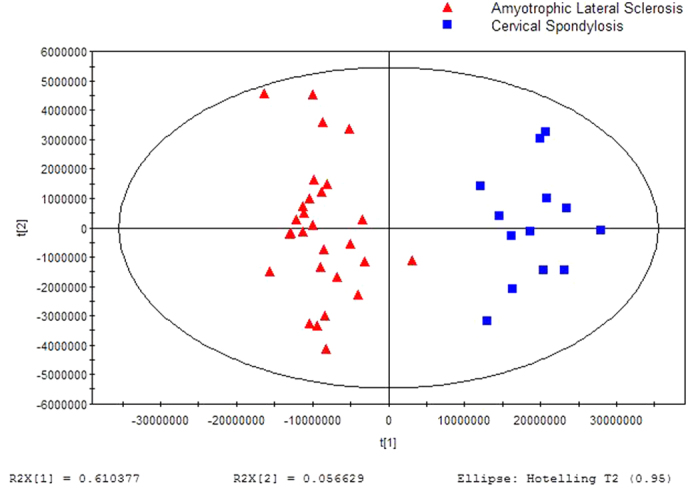
OPLSDA score plot for breath samples from ALS Patients versus CSM Controls: (2 components, R^2^X = 0.667, R^2^Y = 0.911, Q^2^ = 0.895).

**Figure 3:**
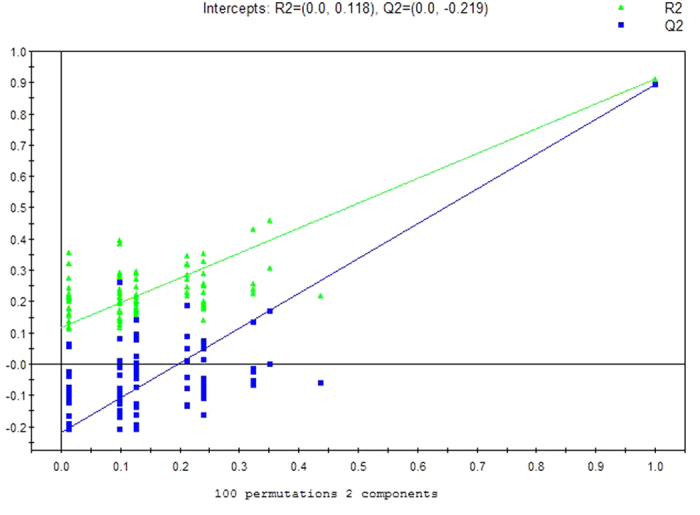
OPLSDA validation plot intercepts for breath samples from ALS Patients versus CSM Controls: R^2^ = (0.0, 0.118); Q^2^ = (0.0, −0.219).

A separation trend was revealed in the two-dimensional PCA score plot (Fig. 1), and the OSC-PLSDA score plot displayed an excellent separation trend between the ALS patients and CSM patients using one predictive component and one orthogonal component (R^2^X = 0.667, R^2^Y = 0.911, and Q^2^ = 0.895) (Fig. 2). Additionally, all of the R^2^ and Q^2^ values calculated from the permutated data were smaller than the original values in the validation plot, which confirmed the validity of the supervised model (Fig. 3).

### Potential Biomarkers

Initially, we found a total of 242 features from the breath samples. Comparing the metabolites obtained by separately processing the data with OPLS-DA or the Kruskal-Wallis test, the 4 metabolites screened out by OPLS-DA were fully included in the 25 compounds screened out by the Kruskal-Wallis test (*p* ＜0.05). This revealed that all of the important information obtained from OPLS-DA was retained. Among the statistically significant metabolites identified using the VIP values in the OSC-PLSDA model and the FDR values, 4 different metabolites were annotated using the NIST 11 database, with a similarity threshold of 75% (Table 2).

### Receiver Operating Characteristic (ROC)

**Figure 4:**
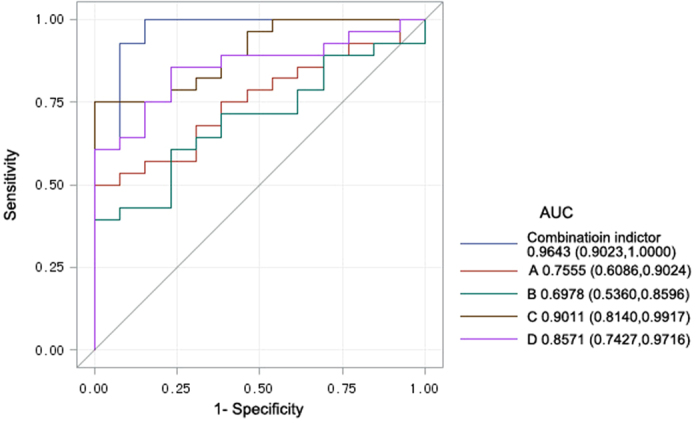
ROC curves of each potential biomarker (A: carbamic acid, monoammonium salt, B: l-alanine ethylamide, (S)-, C: guanidine, N,N-dimethyl-, D: phosphonic acid, (p-hydroxyphenyl)-) and the combined index, with AUC and 95% confidence intervals. The straight line represents an AUC of 0.5.

Both the ROC curves of each potential biomarker and the combined index were graphed to identify the optimal cut-off value (Fig. 4). The Accuracy, Sensitivity, Specificity and AUC of the cut-off point were calculated to evaluate the discriminatory power of the potential biomarkers between ALS and CSM. The statistical analysis of each individual compound with significant differences revealed that the AUC of the ROC curve for carbamic acid, monoammonium salt alone was 0.7555 (95% CI 0.6086–0.9024) with an accuracy of 65.85% (Sensitivity = 0.5, Specificity = 1). The AUC for l-alanine ethylamide, (S)- alone was 0.6978 (95% CI 0.5360–0.8596) with an Accuracy of 58.54% (Sensitivity = 0.393, Specificity = 1). The AUC for Guanidine, N,N-dimethyl- alone was 0.9011 (95% CI 0.8140–0.9917) with an Accuracy of 82.93% (Sensitivity = 0.75, Specificity = 1). The AUC for phosphonic acid, (p-hydroxyphenyl)- alone was 0.8571 (95% CI 0.7427–0.9716) with an Accuracy of 82.93% (Sensitivity = 0.857, Specificity = 0.769). In the analysis of the synthetic biomarkers, the AUC of the ROC curve for the combined index was 0.9643 (95% CI 0.9023–0.1000) with an Accuracy of 92.68% (Sensitivity = 0.929, Specificity = 923) (Table 3).

## Discussion and Conclusion

As a fatal neurodegenerative disease, ALS can cause myasthenia and amyotrophy shortly after the first symptoms emerge. The disease ultimately results in dysarthria and dysphagia as well as respiratory failure, which is the leading cause of death in ALS patients. It has been revealed that the earliest possible intervention to retain the functions of survival motor neurons can improve the prognosis of patients^4^. The symptoms of ALS are similar to the symptoms of CSM. Therefore, this study aimed to explore whether VOC analysis can be used to distinguish ALS from CSM and to identify the potential biomarkers to differentiate between the two.

In this study, we found 4 volatile metabolites that showed significant differences in the exhaled breath between ALS patients and CSM patients, including (1) carbamic acid, monoammonium salt; (2) 1-alanine ethylamide, (S)-; (3) guanidine, N,N-dimethyl-; and (4) phosphonic acid, (p-hydroxyphenyl)-. The SPME-GC-MS method was used to analyze the VOCs, and PCA and OPLSDA were the statistical analysis methods. Compared with the CSM control group, the levels of these compounds in the exhaled breath of ALS patients were all reduced. Additionally, an excellent separation trend was revealed in the OSC-PLSDA score plot. The discriminatory power of the potential biomarkers was measured by AUC using the following definitions: fail–AUC 0.5–0.6, poor–AUC 0.6–0.7, fair–AUC 0.7–0.8, good–AUC 0.8–0.9, and excellent–AUC 0.9–1.0^19^. The AUC of the combined index ROC curve (0.9023–1.0000) revealed that these potential biomarkers have an excellent discriminatory power between ALS and CSM.

While metabolites found in CSF, blood or urine (as discussed in previous literature) and the VOCs identified in our study with SOD1 G93A mice have previously been evaluated, we identified a different panel of metabolites. Biomarkers are particularly attractive for clinical use because they can reflect the specific pathophysiology of the neurodegenerative disorders. The decreased levels of these compounds compared with the CSM control group might be caused by one or more of the following pathological processes: oxidative stress^20^, protein aggregation^21^, excitotoxicity^22^, mitochondrial dysfunction^23^, endoplasmic reticulum stress^24^ and alterations in signaling from astrocytes and microglia^25^,^26^. Oxidative stress, due to a disturbance in the pro- or antioxidant balance, is induced by free radicals or other reactive molecules. The reactive molecules include reactive oxygen species (ROS) and reactive nitrogen species (RNS). In pathological conditions, the unusually elevated concentration of ROS/RNS may result in damage to biological tissues due to the free radical reactions with membrane lipids, nucleic acids, carbohydrates and proteins^27^,^28^. There is an obvious trend in ROS more easily reacting with brain tissues to cause nervous damage owing to the higher oxygen consumption and lipid content.

Currently, the metabolic origin of the VOCs remains uncertain; however, several pathways can be used to explain the decreasing trends. For example, carbamic acid, monoammonium salt, found in the exhaled breath of ALS patients, and diethyldithio-carbamic acid (DETC), are both derivatives of carbamic acid. DETC is a specific inhibitor of superoxide dismutase (SOD)^29^. In the early stages of ALS, the level of SOD is elevated because of oxidative stress. Therefore, it has been hypothesized that there is an increase in synthetic DETC in ALS patients. Subsequently, the level of carbamic acid decreases because it is excessively consumed in DETC synthesis. Finally, a lower concentration of Carbamic acid, monoammonium salt is emitted from the body through breath. Similarly, 1-alanine ethylamide, (S)- and 2-cyano-3,12-dioxooleana-1,9-dien-28-oicacid(CDDO)-ethylamide share an ethylamide group. Neymotin’s study found that CDDO-ethylamide can activate the Nrf2/ARE system (an endogenous cytoprotective system) in NSC-34 G93A SOD1 cells and can extend the lifespan of G93A SOD1 mice^30^. Thus, we speculated that in the early stages of ALS, the compensatory mechanism is initiated and more CDDO-ethylamides are synthesized, which in turn decreases the level of 1-alanine ethylamide, (S)- in exhaled breath. 1,1-Dimethylguanidine is an endogenous inhibitor of nitric oxide (NO) synthesis. Considering the relationship between NO and oxidative stress, the level of NO might be increased in early-stage ALS patients. Under the effects of the compensatory mechanism, the consumption of 1,1-dimethylguanidine increased, which reduced the level of guanidine, N,N-dimethyl- compared with the controls. As for phosphonic acid (p-hydroxyphenyl)-, we found another phosphonic acid derivative, 9-carboxymethyl-4-oxo-5H,10H-imidazo [1,2-a] indeno [1,2-e] pyrazin-2-phosphonic acid (RPR 119990). According to Canton’s research, treatment with RPR 119990, which was found to be active in SOD1 G93A mice, could significantly improve muscle strength and prolong the survival of the mice^31^. This revealed the neuroprotective effects of RPR 119990 in ALS patients, which seems to be a reasonable explanation for the excessive consumption of this compound in early-stage ALS patients. We therefore hypothesized that the phosphonic acid group (which can be used in the synthesis of phosphonic acid, (p-hydroxyphenyl)-) decreased, which further resulted in the level of phosphonic acid, (p-hydroxyphenyl)- decreasing in exhaled breath.

To the best of our knowledge, this is the first time that the VOC analysis of exhaled breath has been applied to screen potential biomarkers for distinguishing ALS from CSM. Applying this analysis is valid for the following reasons: (1) VOC analysis has been used in other neurodegenerative diseases such as Alzheimer’s disease (AD) and Parkinson’s disease (PD)^32^; (2) we previously applied VOC analysis to animal studies of ALS with SOD1 G93A mice; and (3) the design that VOC analysis is used to differentiate ALS and CSM is feasible in theory. Compared with the CSM patients, abnormal metabolism occurred in ALS patients because of different pathogenesis, which inevitably led to the production of specific abnormal metabolites that were different from those of the CSM patients. These metabolites were carried by the circulatory system to the lungs, and the volatile components diffused into the alveoli. The volatile metabolites were then discharged into the exhaled breath through respiration^33^.

In this study, we chose patients with CSM instead of healthy adults as the control group. The reason for this choice is that the symptoms of ALS patients and CSM patients are very similar. Many ALS patients are very likely to be treated as CSM patients; consequently, the most opportune time for effective treatment of ALS patients will be delayed, and the prognosis of patients will be affected. In 2010, Ganesalingam *et al.* revealed that the delay time of ALS patients from the onset of symptoms to confirmed diagnosis was approximately 12–14 months^34^. Therefore, recruiting CSM patients as the control group rather than healthy adults has more clinical significance.

Our study does have some limitations. First and foremost, the metabolic origin of the VOCs is unconfirmed. Secondly, the small sample size meant that the changes in metabolites were not apparent. In addition, our study did not recruit patients who were diagnosed with cervical spinal tumors or syringomyelia (the two other differentiated diseases of ALS) as the control groups, which decreased the specificity of the potential biomarkers we obtained. Therefore, further studies should include a larger sample size and should recruit patients with cervical spinal tumors or syringomyelia. In conclusion, we identified 4 potential volatile biomarkers in the exhaled breath of ALS patients that showed significant differences compared with CSM patients. While the potential molecular mechanism underlying VOC production remains unclear, it is clear that breath analysis has a potential clinical application in distinguishing ALS from the ALS-mimicking disease CSM and may provide a better understanding of the underlying pathophysiological mechanisms of ALS.

## Additional Information

**How to cite this article**: Wang, C. *et al.* Comparative Analysis of VOCs in Exhaled Breath of Amyotrophic Lateral Sclerosis and Cervical Spondylotic Myelopathy Patients. *Sci. Rep.* **6**, 26120; doi: 10.1038/srep26120 (2016).

## Figures and Tables

**Table 1:** ALS-CSM patient information analysis.

		CSM	ALS	P-value
Age		53.5(8.13)	58.6(12.54)	0.182
Sex	Male (n)	6	18	0.273
	Female (n)	7	10	
Total (n)		13	28	

Abbreviations: CSM, cervical spondylotic myelopathy; ALS, amyotrophic lateral sclerosis.

**Table 2:** Potential biomarkers.

Potential biomarker	P-value	FC	VIP	FDR
carbamic acid, monoammonium salt	0.00917	0.192485	14.8788	0.02769
l-alanine ethylamide, (S)-	0.04368	0.104253	1.02574	0.09202
guanidine, N,N-dimethyl-	0.00004	0.660394	2.41899	0.00049
phosphonic acid, (p-hydroxyphenyl)-	0.00027	0.104253	1.62293	0.00170

Abbreviations: VIP, variable importance in the projection, FC, fold change, defined as: FC = log10(X2/X1), where X1 denoted the arithmetic mean value of a certain metabolite in the case group and X2 denoted the arithmetic mean value in the control group. FC with a positive value indicates that the concentration of a certain metabolite is relatively higher in CSM patients controls compared with ALS patients; FDR, false discovery rate.

**Table 3:** The Accuracy, Sensitivity, Specificity and AUC of each potential biomarker and the combined index.

Biomarker	Accuracy	Sensitivity	Specificity	AUC
carbamic acid, monoammonium salt	65.85%	0.5	1	0.7555
l-alanine ethylamide, (S)-	58.54%	0.393	1	0.6978
guanidine, N,N-dimethyl-	82.93%	0.75	1	0.9011
phosphonic acid, (p-hydroxyphenyl)-	82.93%	0.857	0.769	0.8571
combined index	92.68%	0.929	0.923	0.9643

Abbreviations: AUC, area under the ROC curve.
